# Analgesic Effect of Acetaminophen: A Review of Known and Novel Mechanisms of Action

**DOI:** 10.3389/fphar.2020.580289

**Published:** 2020-11-30

**Authors:** Nobuko Ohashi, Tatsuro Kohno

**Affiliations:** ^1^ Division of Anesthesiology, Niigata University Graduate School of Medical and Dental Sciences, Niigata, Japan; ^2^ Department of Anesthesiology and Intensive Care Medicine, International University of Health and Welfare School of Medicine, Narita, Japan

**Keywords:** acetaminophen, *N*-acylphenolamine, analgesia, brain, spinal dorsal horn

## Abstract

Acetaminophen is one of the most commonly used analgesic agents for treating acute and chronic pain. However, its metabolism is complex, and its analgesic mechanisms have not been completely understood. Previously, it was believed that acetaminophen induces analgesia by inhibiting cyclooxygenase enzymes; however, it has been considered recently that the main analgesic mechanism of acetaminophen is its metabolization to *N*-acylphenolamine (AM404), which then acts on the transient receptor potential vanilloid 1 (TRPV1) and cannabinoid 1 receptors in the brain. We also recently revealed that the acetaminophen metabolite AM404 directly induces analgesia via TRPV1 receptors on terminals of C-fibers in the spinal dorsal horn. It is known that, similar to the brain, the spinal dorsal horn is critical to pain pathways and modulates nociceptive transmission. Therefore, acetaminophen induces analgesia by acting not only on the brain but also the spinal cord. In addition, acetaminophen is not considered to possess any anti-inflammatory activity because of its weak inhibition of cyclooxygenase (COX). However, we also revealed that AM404 induces analgesia via TRPV1 receptors on the spinal dorsal horn in an inflammatory pain rat model, and these analgesic effects were stronger in the model than in naïve rats. The purpose of this review was to summarize the previous and new issues related to the analgesic mechanisms of acetaminophen. We believe that it will allow clinicians to consider new pain management techniques involving acetaminophen.

## Introduction

Acetaminophen is one of the most commonly used analgesic agents for alleviating acute and chronic pain. Due to its safety, acetaminophen is prescribed for patients in whom non-steroidal anti-inflammatory drugs (NSAIDs) are contraindicated, such as those with gastric ulcers and bronchial asthma, pregnant women, nursing mothers, and children (Leung, 2012; Roberts et al., 2016). It has also been placed on all three steps of pain treatment intensity of the WHO analgesic ladder for the treatment of cancer pain. However, its metabolism is complex, and its analgesic mechanisms have not been completely understood. Previously, it was thought that acetaminophen induces analgesia by inhibiting the enzyme cyclooxygenase (COX), but now it is believed that acetaminophen is metabolized to *p*-aminophenol, which crosses the blood-brain barrier and gets metabolized by fatty acid amide hydrolase to yield *N*-acylphenolamine (AM404). AM404 acts on the transient receptor potential vanilloid 1 (TRPV1) and cannabinoid 1 (CB1) receptors in the midbrain and medulla (Roberts et al., 2002; Jennings et al., 2003; Mallet et al., 2010), which are co-localized mediators of pain modulation (De Petrocellis et al., 2000; Palazzo et al., 2002; Maione et al., 2006). Therefore, acetaminophen induces analgesia via direct action on the brain (Bannwarth et al., 1992; Gelgor et al., 1992; de Lange et al., 1994; Hogestatt et al., 2005), and these receptor sites on the brain are the main mediators of acetaminophen-induced analgesia. However, our group recently revealed a new analgesic mechanism of acetaminophen, using behavioral measures, *in vivo* and *in vitro* whole-cell patch-clamp recordings with rats, wherein the acetaminophen metabolite AM404 directly induces analgesia via TRPV1 receptors on the spinal dorsal horn (Ohashi et al., 2017). Similar to the brain, the spinal cord, especially substantia gelatinosa (SG, lamina II of Rexed), is also critical to pain pathways, and modulates nociceptive transmission via primary afferent Aδ- and C-fibers (Kohno et al., 1999; Ohashi et al., 2017). Furthermore, TRPV1 receptors are abundant in the spinal cord dorsal horn (Yang et al., 1998; Yang et al., 1999; Yang et al., 2000). Therefore, our results describing the new analgesic mechanism underlying the action of acetaminophen on the spinal dorsal horn, are reasonable compared to previous reports (Ohashi et al., 2017).

Acetaminophen does not possess any anti-inflammatory activity, because it is a very weak inhibitor of COX and does not inhibit neutrophil activation (Hanel and Lands, 1982). Therefore, even though it has always been discussed together with NSAIDs in terms of pharmacological mechanism, acetaminophen is not regarded as an NSAID and is not appropriate for treating inflammatory pain conditions. However, we also revealed that acetaminophen metabolite AM404 induces analgesia via TRPV1 receptors on the spinal dorsal horn in a rat model of inflammatory pain, and these analgesic effects were stronger in the inflammatory pain model than in naïve rats (Ohashi et al., 2017).

The purpose of this review was to summarize the previous and new issues related to the analgesic mechanisms of acetaminophen and discuss our understanding that acetaminophen metabolite AM404 also acts on the spinal dorsal horn and induces analgesia in inflammatory pain conditions. This review will allow clinicians to consider new pain management techniques using acetaminophen.

## Analgesic Mechanisms of Acetaminophen

### Inhibition of Cyclooxygenase Activity

**FIGURE 1:**
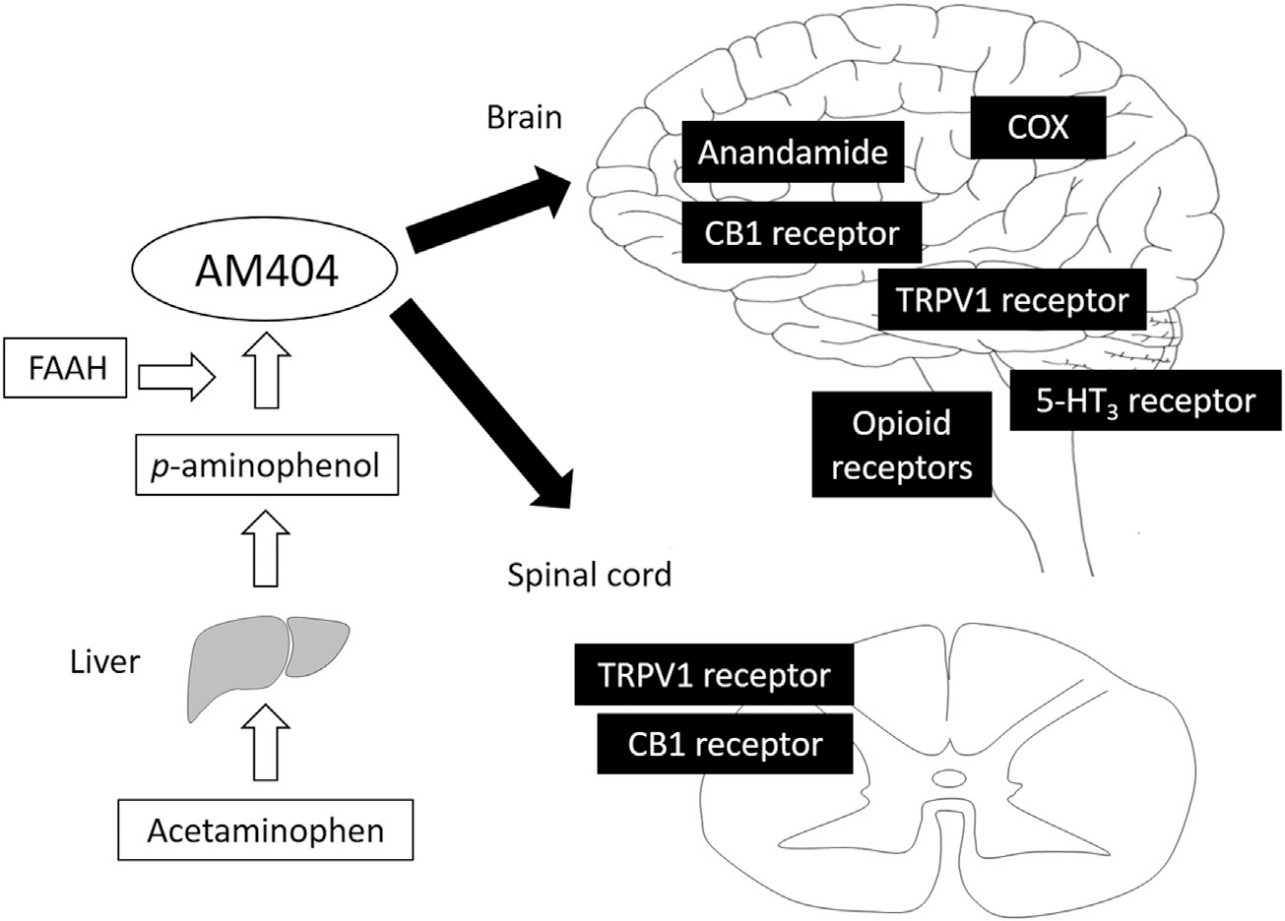
Analgesic mechanism of acetaminophen. Acetaminophen is metabolized to *p*-aminophenol, which easily crosses the blood-brain barrier and is converted to AM404 by FAAH. AM404 mainly acts on both the brain and spinal cord via COX, anandamide, CB1, TRPV1, opioid, and 5-HT3 receptors. AM404, N-acylphenolamine; FAAH, fatty acid amide hydrolase; COX, cyclooxygenase; CB1, cannabinoid 1; TRPV1, transient receptor potential vanilloid 1.

It has been thought that acetaminophen induces analgesia by blocking prostaglandin synthesis from arachidonic acid by inhibiting the enzymes, COX-1 and -2. However, unlike NSAIDs, acetaminophen interferes with the peroxidase activity of COX isoenzymes, predominantly COX-2, with little clinical effect and depends to a great extent on the state of environmental oxidation (Graham et al., 2013; Aminoshariae and Khan, 2015). It has also been reported that the third COX isoenzyme, COX-3, which is an exon splice variant of COX-1, is especially sensitive to acetaminophen (Chandrasekharan et al., 2002). However, it soon appeared that COX-3 is not found in humans, and further studies suggest that acetaminophen has no clinically significant effects on the COX-1 exon splice variants found in humans so far (Graham and Scott, 2005). It is now considered that the inhibition of COX activity is not the main analgesic mechanism of acetaminophen (Table 1; Figure 1).

**TABLE 1:** Analgesic mechanism of acetaminophen.

Medicine	Target site	Effect/mechanism	References
Acetaminophen	COX-1, COX-2	Inhibitory	Aminoshariae and Khan (2015)
Acetaminophen	COX-2	Inhibitory	Graham et al. (2013)
Acetaminophen	COX-3	Inhibitory	Chandrasekharan et al. (2002)
Acetaminophen	COX-3	No clinically relevant effects	Graham and Scott (2005)
NAPQI	TRPA1	Activating	Andersson et al. (2011)
Acetaminophen	TRPA1	Activating	Gentry et al. (2015)
AM404	Anandamide transport inhibitor, CB1 receptor	Re-uptake inhibitor, Activating	Beltramo et al. (1997)
AM404	TRPV1 receptor	Activating	Zygmunt et al. (2000), Hogestatt et al. (2005), Roberts et al. (2002), Jennings et al. (2003), Mallet et al. (2010), Barrière et al. (2013),
AM404	CB1 receptor, TRPV1 receptor	Activating	Rodella et al. (2005), Borsani et al. (2007)
AM404	CB1 receptor, TRPV1 receptor	Not activating, Activating	Ohashi et al. (2017)
AM404	CB1 receptor < TRPV1 receptor	Activating	Szallasi and Di Marzo (2000)
Acetaminophen	Opioids	Activating	Raffa et al. (2000), Raffa et al. (2004)
Acetaminophen	Serotonin	Increases content	Pini et al. (1996)
Acetaminophen	5-HT_3_ receptor	Activating	Alloui et al. (2002), Pickering et al. (2006), Pickering et al. (2008)

NAPQI, *N*-acetyl-*p*-benzoquinoneimine; AM404, *N*-acylphenolamine; COX, cyclooxygenase; TRPA1, transient receptor potential ankyrin 1; CB1, cannabinoid 1; TRPV1, transient receptor potential vanilloid 1.

### Activating the Transient Receptor Potential Vanilloid 1 and Cannabinoid 1 Receptors

Acetaminophen is first metabolized to *p*-aminophenol, which easily crosses the blood-brain barrier and is converted to AM404 by fatty acid amide hydrolase (Hogestatt et al., 2005). Acetaminophen is also metabolized to other compounds through another pathway, such as *N*-acetyl-*p*-benzoquinoneimine (NAPQI), which also appears to produce analgesia by activating transient receptor potential ankyrin 1 receptors (Andersson et al., 2011; Gentry et al., 2015). However, AM404 is widely known to be the most important mediator of acetaminophen metabolite-induced analgesia. Although AM404 was thought to be just an anandamide analog which acts on CB1 receptors (Beltramo et al., 1997), it was recently shown that AM404 also acts on TRPV1 receptors (Zygmunt et al., 2000; Hogestatt et al., 2005; Barrière et al., 2013). In particular, it is known that TRPV1 receptors in the brain are important for pain modulation. Two examples involving TRPV1 receptors are cannabidiol, the primary nonaddictive component of cannabis, which induces analgesia through TRPV1 receptor activation in the dorsal raphe nucleus (De Gregorio et al., 2019), and dipyrone, an antipyretic and non-opioid analgesic drug which causes analgesia by acting on TRPV1 and CB1 receptors in rostral ventromedial medulla (Maione et al., 2015). Therefore, it is now considered that AM404 acts on TRPV1 receptor in the brain and induces analgesia. For example, by activating TRPV1 receptor, AM404 produced outward currents that were measured using whole-cell patch-clamp recordings and acted as a partial agonist in trigeminal neurons (Roberts et al., 2002; Jennings et al., 2003). Moreover, intracerebroventricular injection of AM404 produced analgesia in the formalin test (Mallet et al., 2010). Therefore, these receptors in the brain are widely considered to be the main mediators of acetaminophen-induced analgesia. They are also the reason why acetaminophen exhibits a “central” effect for long periods.

Similar to the brain, it is also known that the spinal cord, especially SG neurons, is critical to pain pathways, and modulates nociceptive transmission via primary afferent Aδ- and C-fibers (Kohno et al., 1999; Ohashi et al., 2015). Furthermore, it is also known that TRPV1 and CB1 receptors are abundant in the spinal cord dorsal horn (Yang et al., 1998; Yang et al., 1999; Yang et al., 2000). Therefore, there is a possibility that, in addition to its actions in the brain, acetaminophen and/or its metabolite AM404 also induce analgesia via direct activation of TRPV1 and/or CB1 receptors in the spinal cord dorsal horn. In fact, a few previous studies have shown that AM404 decreases neuronal *c-fos*-positive immunoreactivity induced by non-noxious stimulation of the spinal cord in a rat model of neuropathic or inflammatory pain, and these responses are inhibited by TRPV1 or CB1 receptor antagonists (Rodella et al., 2005; Borsani et al., 2007). Nevertheless, the precise analgesic mechanisms of acetaminophen in the spinal cord via its AM404 metabolite are still unknown, because previous studies have not examined the synaptic transmission at the cellular level. Therefore, it was believed that acetaminophen does not act on the spinal cord. However, our group recently revealed a new analgesic mechanism of acetaminophen, using behavioral measures, and *in vivo* and *in vitro* whole-cell patch-clamp recordings with naïve rats (Ohashi et al., 2017). We first demonstrated with behavioral experiments that intraperitoneal injections of acetaminophen and intrathecal injections of AM404 induce analgesia to thermal stimulation. We next conducted *in vivo* and *in vitro* whole-cell patch-clamp recordings of SG neurons in the spinal cord dorsal horn and recorded the excitatory post-synaptic currents (EPSCs). With *in vivo* patch-clamp recording, the areas under the curve, which is surrounded by the baseline and border of the EPSCs, were significantly reduced after intravenous injection of acetaminophen following peripheral pinch stimuli. However, with *in vitro* patch clamp recording, direct application of acetaminophen to the spinal cord did not change miniature EPSCs (mEPSCs), but AM404 did. These results suggest that systemic administration of acetaminophen metabolizes to AM404, which directly acts on spinal cord dorsal horn and induces analgesia. We also examined the effects of AM404 on EPSCs evoked from primary afferent neurons by stimulating the dorsal root and demonstrated that AM404 reduces the amplitudes of monosynaptic EPSCs evoked by stimulating C-fibers, but not Aδ-fibers. These responses were inhibited by the TRPV1 receptor antagonist, but not CB1 receptor antagonist. Therefore, we found that acetaminophen was metabolized to AM404, which induces analgesia by directly inhibiting the excitatory synaptic transmission via TRPV1 receptors expressed on terminals of C-fibers in the spinal dorsal horn. Contrary to previous studies on the brain, we failed to find the analgesic effect of acetaminophen/AM404 on the CB1 receptor on spinal dorsal horn neurons. We believe that the main reason for the differences between our results and that of previous reports was the concentration of AM404 (30 µM) in our study, which is equivalent to the clinically recommended dosage of acetaminophen (20 mg/kg). Szallasi *et al.* compared the affinities of AM404 for brain TRPV1 and CB1 receptors and reported that the concentration of AM404 required to activate TRPV1 receptors is much lower than that required for CB1 receptors (Szallasi and Di Marzo, 2000). Therefore, there is a possibility that the concentration of AM404 in our study was insufficient to activate CB1 receptors in dorsal horn neurons and higher doses of AM404 may also act on the CB1 receptor in the spinal dorsal cord. We believe that our new analgesic mechanism of acetaminophen will contribute to the development of new techniques for clinical pain management using acetaminophen.

### Other Mechanisms

Another possible reason for the analgesic action of acetaminophen could be the action of endogenous neurotransmitter systems including opioid and serotonergic systems. Previous studies have reported that the analgesic effect of acetaminophen involves the recruitment of endogenous opioid pathways that lead to analgesic spinal-supraspinal self-synergy (Raffa et al., 2000), and the analgesic effects induced by intrathecal injection or intracerebroventricular injection of acetaminophen were attenuated by *mu*-, *delta*-, and *kappa*-opioid receptor antagonists (Raffa et al., 2004). This analgesic self-synergy is significantly attenuated by the administration of naloxone, an opioid receptor antagonist, at the spinal level (Raffa et al., 2000). Similarly, another study reported that depletion of brain serotonin prevented the analgesic effect of acetaminophen in the hot-plate test and in the first phase of the formalin response. Furthermore, acetaminophen significantly increased the serotonin content in the pontine and cortical areas (Pini et al., 1996). It is also reported that the serotonin receptor has several subtypes, and acetaminophen-induced analgesia was inhibited by intrathecal or intravenous injection of tropisetron, a 5 hydroxytryptamine_3_ (5-HT_3_) receptor antagonist (Alloui et al., 2002; Pickering et al., 2006; Pickering et al., 2008). These findings implied that acetaminophen may be involved in endogenous opioid or descending serotonergic pathways as contributors to the analgesic action of acetaminophen.

### Analgesic Effect of Acetaminophen for Inflammatory Pain

For many decades, acetaminophen was not considered to possess any anti-inflammatory activity and was, therefore, not appropriate for treating allodynia or hyperalgesia in inflammatory pain conditions. A study has reported that acetaminophen is a very weak inhibitor of COX, which does not inhibit neutrophil activation (Hanel and Lands, 1982). For example, at the therapeutic concentration, acetaminophen inhibits COX activity when the levels of arachidonic acid and peroxide are low but has little effect when the levels of arachidonic acid or peroxide are high as seen in severe inflammatory conditions such as rheumatoid arthritis (Hanel and Lands, 1982). However, our group also revealed that acetaminophen metabolite AM404 induces analgesia in rats of the inflammatory pain model (Ohashi et al., 2017). Similar to the results in naïve rats, our behavioral studies in an inflammatory pain rat model suggest that acetaminophen and AM404 induce analgesia to thermal stimulation. Moreover, both *in vivo* and *in vitro* whole-cell patch-clamp recordings have shown that acetaminophen metabolite AM404 directly inhibits excitatory synaptic transmission via TRPV1 receptors expressed on terminals of C-fibers in the spinal dorsal horn. Moreover, analgesic effects induced by acetaminophen and AM404 in the rats used for the inflammatory pain model were stronger than those in naïve rats (Ohashi et al., 2017). It is known that there is an increased proportion of TRPV1-protein-positive neurons during inflammation in dorsal root ganglion and unmyelinated axons of the digital nerves (Carlton and Coggeshall, 2001). Therefore, increased TRPV1 activity in the rats used for the inflammatory pain model suggests strong analgesic effects following acetaminophen and AM404 administration. Therefore, our findings are consistent with previous research, and we believe that our results will allow clinicians to consider new pain management techniques involving acetaminophen.

## Pharmacokinetics and Side Effects

When the appropriate dosage of acetaminophen is used, serious side effects seldom occur; however, some case studies have reported liver toxicity caused by acetaminophen. Usually, acetaminophen is administered orally or intravenously. The maximum single-dose of acetaminophen for the treatment of pain or fever is 1,000 mg every 4 h as needed, up to a recommended maximum daily dose of 4 g. These therapeutic concentrations range from 5 to 20 mg/ml. Acetaminophen has a very high oral bioavailability of 60–88% (Bertolini et al., 2006), and after oral administration of 1,000 mg acetaminophen, the plasma maximum concentration (*C*_max_) is 12.3 μg/ml, area under the curve over 6 h (AUC_0–6_) is 29.4 μg/h/ml, and AUC extrapolated to infinity (AUC_0–∞_) is 44.4 μg/h/ml. The time to maximal concentration (*T*_max_) is 1.0 h, and the elimination half-life (*t*_1/2_) is 2.53 h. In contrast, after intravenous administration of 1,000 mg acetaminophen, the plasma *C*_max_ is 21.6 μg/ml, AUC_0–6_ is 42.5 μg/h/ml, and AUC_0–∞_ is 50.0 μg/h/ml. The *T*_max_ is 0.25 h, and the *t*_1/2_ is 2.17 h (Singla et al., 2012). These findings suggest that intravenous administration of acetaminophen shows earlier and higher peak plasma levels than oral administration; however, there is no difference in AUC and *t*_1/2_ between the intravenous and oral administration.

Once acetaminophen metabolizes in the liver by conjugation with glucuronic acid (40–67%), sulfuric acid (20–46%), and *p*-aminophenol, it easily crosses the blood-brain barrier and is converted to AM404 (Gazzard et al., 1973; Duggin and Mudge, 1975). Furthermore, about 5% of acetaminophen is subjected to *N*-hydroxylation in the liver with the involvement of cytochrome P450 enzymes (especially CYP2E1) to form the toxic metabolite, NAPQI (Bertolini et al., 2006). Normally, NAPQI is detoxified into harmless metabolites via conjugation of the sulfhydryl groups of glutathione by glutathione S-transferase into mercapturic acid, which is eliminated in the urine (Mitchell et al., 1974; Potter et al., 1974; Benson et al., 2005; Bertolini et al., 2006). However, glutathione can become depleted after overuse of acetaminophen or in cases of weakened hepatic function (caused by slimming, malnutrition, hepatitis C virus, or alcohol overuse), which causes accumulation of NAPQI. When this happens, NAPQI interacts covalently with liver cell components resulting in hepatic damage. To detoxify the liver toxicity caused by NAPQI, *N*-acetylcysteine must be ingested as soon as possible.

Usually, acetaminophen is administered by oral, transanal, and intravenous routes, and NAPQI is produced by acetaminophen during the metabolic pathways. However, we think that if we administer AM404 instead of acetaminophen using intrathecal or intracerebroventricular injection, we could observe a stronger analgesic effect with reduced side effects at a smaller dosage. Therefore, further clinical studies on the effectiveness and safety of acetaminophen will be needed.

## Conclusion

Acetaminophen acts not only on the brain but also the spinal cord and induces analgesia. Moreover, the most possible analgesic mechanism is that the acetaminophen metabolite AM404 acts by activating TRPV1 and/or CB1 receptors. Our data also support a mechanism by which acetaminophen also induces analgesia in inflammatory pain conditions. These findings are applicable to clinical pain management with acetaminophen, but the analgesic mechanism of acetaminophen has not been elucidated completely. Therefore, further discussions and studies will be needed to understand the action of acetaminophen.

## Author Contributions

All authors listed have made a substantial, direct, and intellectual contribution to the work and approved it for publication.

## Conflict of Interest

The authors declare that the research was conducted in the absence of any commercial or financial relationships that could be construed as a potential conflict of interest.
